# Cost-effectiveness of hand hygiene promotion for MRSA blood stream infection in ICU settings

**DOI:** 10.1186/2047-2994-4-S1-O50

**Published:** 2015-06-16

**Authors:** N Luangasanatip, M Hongsuwan, Y Lubell, D Limmathurotsakul, P Srisamang, NPJ Day, N Graves, BS Cooper

**Affiliations:** 1 Mahidol-Oxford Research Unit, Bangkok, Thailand; 2 Pediatrics, Sappasithiprasong Hospital, Ubon Ratchatani, Thailand; 3 School of Public Health, Queensland University of Technology, Brisbane, Australia

## Introduction

Multimodal interventions are effective in increasing hand hygiene compliance amongst healthcare workers, but it is not known whether such interventions are cost-effective outside high-income countries.

## Objectives

To determine whether reductions in Methicillin-resistant *Staphylococcus aureus* bloodstream infections (MRSA-BSI) alone would make hand hygiene interventions cost-effective in intensive care units (ICUs) in a middle-income country using a model-based framework.

## Methods

Transmission dynamic and decision analytic models were combined to determine the expected impact of hand hygiene interventions on MRSA-BSI incidence and evaluate their cost-effectiveness. Epidemiological and economic parameters were derived using data from a tertiary hospital in North-east Thailand. Sensitivity analyses were performed with different values for MRSA transmissibility and colonization prevalence on admission.

## Results

Interventions increasing hand hygiene compliance from a 10% baseline to ≥20% are likely to be cost-effective solely through reduced MRSA-BSI. Increasing compliance from 10% to 40% was estimated to cost $US 89·1 per bed-year with 4·07 QALYs gained per 10,000 bed-days in the paediatric ICU (PICU) and $US 63·3 per bed-year with 4·03 QALYs gained per 10,000 bed-days in the adult ICU. If baseline compliance is not greater than 20%, the intervention is always cost-effective even with only a 10% compliance improvement.

## Conclusion

Effective multimodal hand hygiene interventions are likely to be cost-effective in ICU settings in typical middle-income countries where baseline compliance is low due to preventing MRSA-BSI alone. Where compliance is higher, the cost-effectiveness of interventions to improve it further will depend on the impact on HAIs other than MRSA-BSI.

## Disclosure of interest

None declared.

